# Covalent Immobilization of Crown Ether on Cellulose Acetate Membranes for Enhanced Heavy Metal Ion Retention

**DOI:** 10.3390/polym18111371

**Published:** 2026-05-31

**Authors:** Eduard Ionut Piscanu, Andreea Madalina Pandele, Madalina Oprea, Adrian Ionut Nicoara, Stefan Ioan Voicu

**Affiliations:** 1 Department of Analytical Chemistry and Environmental Engineering, Faculty of Chemical Engineering and Biotechnologies, National University of Science and Technology POLITEHNICA Bucharest, 1-7 Gheorghe Polizu, 011061 Bucharest, Romania; eduard.piscanu@yahoo.com (E.I.P.); pandele.m.a@gmail.com (A.M.P.); madalina.calarasu@upb.ro (M.O.); 2 Advanced Polymer Materials Group, National University of Science and Technology POLITEHNICA Bucharest, 1-7 Gheorghe Polizu, 011061 Bucharest, Romania; 3 Department of Science and Engineering of Oxide Materials and Nanomaterials, Faculty of Chemical Engineering and Biotechnologies, National University of Science and Technology POLITEHNICA Bucharest, 1-7 Gheorghe Polizu, 011061 Bucharest, Romania; adi.nicoara18@gmail.com; 4 Academy of Romanian Scientists, 54 Splaiul Independentei Street, District 5, 050094 Bucharest, Romania

**Keywords:** cellulose acetate, crown ethers, heavy metal ions retention

## Abstract

Heavy metal contamination in water remains a major environmental concern due to the persistence, toxicity, and bioaccumulation potential of metal ions such as Ni^2+^ and Cu^2+^. Therefore, the development of sustainable membrane materials with improved permeability and metal ion retention capacity is of significant interest for advanced water purification applications. In this research, crown ether-functionalized cellulose acetate membranes were developed by employing cyanuric chloride as a linker in order to enable advanced heavy metal ion retention capacity. In order to achieve this, the modification process involved a multi-step approach comprising successive hydroxylation, silanization, triazine activation, and crown ether grafting. The successful functionalization was confirmed by FTIR (Fourier Transform Infrared Spectroscopy) and XPS (X-ray Photoelectron Spectroscopy) analyses, while thermal characterization demonstrated improved stability over a wide range of temperatures without compromising the integrity of the cellulose acetate backbone. The crown-ether-functionalized membranes exhibited enhanced performance in terms of heavy metal ion separation, demonstrating significantly higher retention of Ni^2+^ (30%) and Cu^2+^ (27%) as compared to pristine CA membranes (<10%) over repeated filtration cycles. These results demonstrate that crown ether functionalization is a versatile approach for tuning the interfacial features of cellulose acetate membranes in order to achieve increased permeability and selectivity toward heavy metal removal, highlighting their potential for advanced water purification applications.

## 1. Introduction

Access to clean and safe water is fundamental for human health and environmental sustainability. Over the last decades, the considerable increase in industrial activities, mining, and urbanization have led to an extensive contamination of water resources by heavy metals [1,2,3,4,5,6]. Various ions such as copper (Cu^2+^) [7,8], nickel (Ni^2+^) [9,10], and other transition metals [11,12] can accumulate in aquatic systems and subsequently in living organisms, leading to harmful effects to both environment and health even at low concentrations. According to drinking water guidelines, the limit value for Ni is 0.07 mg/L, while Cu is commonly regulated at 2 mg/L in drinking water. Concentrations above these value may pose risks depending on the exposure time and water chemistry [13]. Thus, it is vital to remove or manage such contaminants by employing advanced purification techniques [14] in order to maintain extremely low concentrations in water sources.

Several methods have been developed for the removal of heavy metal ions from contaminated water sources including coagulation, flocculation [15,16,17], chemical precipitation, ion exchange, electrochemical treatments and membrane-based separation. Chemical precipitation is one of the most used techniques at the industrial scale due to its simplicity and capacity to treat large volumes. However, it often generates sludge, and its efficiency decreases at low metallic ions concentration. Adsorption is a strong alternative due to its ease of operation and potential use at low contaminant concentration, but its performance is strongly influenced and depends on sorption capacity, regeneration and mass-transfer limitations. On the other hand, coagulation and flocculation can remove particle and colloids but these techniques imply the use of specific reagents and additional post-treatment operations. Ion exchange is another technique with high selectivity and good removal efficiency, although the costs associated with the resin and regeneration as well as sensitivity to competing ions may limit its applications. Membrane-based purification processes provide continuous separation and compact operation, although several parameters such as fouling, permeability and material stability remain one of the most important challenges imposed by this technique [18,19]. In this context, the functionalization of polymeric membranes represents an important approach through which advanced filtration performance and specific binding interactions can be achieved. Therefore, the need for advanced purification technologies that can efficiently target and remove heavy metals is imperative.

Cellulose acetate is one of the most used polymeric materials for membrane fabrication due to its good film-forming ability, low cost, moderate hydrophilicity, and accessibility [20,21,22,23]. Cellulose acetate membranes exhibit favorable mechanical properties, chemical stability, and ease of processing. These properties make it an attractive candidate for water treatment and separation applications such as desalination, ultrafiltration, and pollutant removal [24,25,26,27]. Additionally, its abundant ester and hydroxyl functionalities provide valuable sites for further surface modification, enabling the introduction of specific chemical functionalities without altering the bulk membrane structure [28,29]. However, when it comes to specific applications, pristine cellulose acetate membranes generally lack selectivity toward precise metal ions, which limits their effectiveness in advanced water purification applications. Consequently, surface functionalization strategies are necessary to enhance their affinity and selectivity for targeted contaminants, such as heavy metal ions.

Crown ethers (CE) are well-known macrocyclic ligands with an ether (oxygen–carbon–carbon) ring structure and multiple electron-donating oxygen atoms [30,31]. Their distinct architecture favors strong and selective complexation of metal ions, making them attractive candidates for specific separation and removal applications [32,33,34]. When grafted onto the surface of polymeric membranes, crown ether moieties combine supramolecular chemistry with membrane filtration, enhancing metal ion retention without influencing permeability [35,36]. Previous studies on polymeric membranes functionalized with crown ether [32,37,38,39] have shown superior results in terms of ion selectivity, transport efficiency, and chemical stability. These characteristics make crown ether-functionalized membranes of particular interest for efficient and selective heavy-metal ion removal from water systems.

Recent work on crown ether-based nanofiltration membranes has demonstrated that crown ether functionalities can significantly improve selective ion separation performance [40,41,42]. For instance, a polyamide membrane modified with diazo-18-crown-6 ether exhibited strong resistance to Mg^2+^ transport while favoring Li^+^ permeation achieving a Mg^2+^ rejection of 87.7% and an Mg^2+^/Li^+^ separation factor up to 18 [35]. Retention of Li+ ions up to 5.5 mg/g was achieved when crown ethers were integrated into a hydrogel that developed for lithium recovery from salt-lake brine [43].

Du et al. [33] developed 18-crown-6 ether-functionalized polyimide nanofiber membranes synthesized by in situ grafting and electrospinning. The resulting membranes showed a higher Cs^+^ adsorption capacity (~85.2 mg g^−1^) as compared with unmodified membranes (~68.2 mg g^−1^) and excellent selectivity over competing ions, with separation factors up to 29.5. Simulation studies confirmed that the concentration of crown ether influences Cs^+^ retention through strong ion–dipole interactions, highlighting the effectiveness of macrocyclic coordination sites for selective ion capture.

In this context, the present study introduces a stepwise functionalization approach for cellulose acetate membranes, in which crown ether moieties are covalently immobilized to improve the selective retention of heavy metal ions while preserving the integrity of the membrane. In this study, Ni^2+^ and Cu^2+^ ions were selected as representative divalent heavy metal contaminants to evaluate the retention performance of the developed membranes. These ions were chosen due to their environmental relevance and their ability to coordinate with oxygen- and nitrogen-containing functional groups, including those present in crown ether-functionalized structures.

Although crown ether-containing materials have been previously explored for the adsorption or complexation of metal ions, their covalent immobilization onto cellulose acetate membranes through a cyanuric chloride-mediated functionalization route remains less explored. The novelty of the present work lies in the design of a bio-based membrane platform in which crown ether moieties are chemically anchored onto the cellulose acetate surface, allowing the combination of membrane filtration properties with metal ion-recognition functionality. Unlike conventional adsorbent systems, this approach aims to improve both permeability and heavy metal ion retention by tailoring the interfacial chemistry of the membrane.

## 2. Materials and Methods

### 2.1. Materials

Commercial Cellulose Acetate membranes (diameter 47 mm, porosity 0.45 μm) from Prat Dumas France were used as received. Sodium hydroxide 98% (NaOH), Ethanol, (3-Aminopropyl) triethoxysilane ≥ 98.0% (APTS), Cyanuric chloride 99% (CN) and 4′-Aminobenzo-15-crown-5 ether 97% (CE) from Sigma Aldrich (St. Louis, MO, USA) were used without further purification. For the heavy metal retention tests, Nickel(II) acetate tetrahydrate 98% (Ni(C_2_H_3_O_2_)_2_ × 4H_2_O) and Copper(II) sulfate pentahydrate (CuSO_4_ × 5H_2_O), ACS reagent, ≥98.0% from Sigma Aldrich were employed. The water utilized in all experiments was distilled water.

### 2.2. Chemical Modification of Cellulose Acetate Membranes Surface with CE

**Figure 1:**
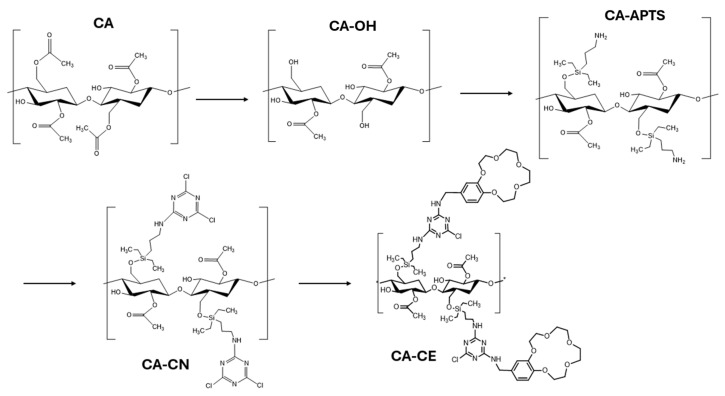
Schematic representation of the main steps involved in the synthesis of crown ether-modified CA membranes.

The grafting of crown ether moieties onto a CA membrane surface was performed by following a multi-step protocol adapted from ref. [44]. The theoretical mechanism of the functionalization process is schematically represented in Figure 1.

The first step of the functionalization process of the CA membrane surface consisted of a partial deacetylation to introduce hydroxyl groups. Thus, CA membranes were reacted for 24 h at room temperature with 5% NaOH (20 mL NaOH solution per membrane). After the synthesis, the membranes were washed with distilled water until neutral pH and dried. The deacetylated CA membranes will further be referred to as CA-OH. Then, the free OH groups from CA-OH were reacted with APTS by immersing them in 20 mL mild basic solution per membrane (2 mL of 0.1 N NaOH per membrane) of 20% APTS in ultra-pure water for 24 h at 37 °C. After the completion of the reaction, membranes were washed with distilled water to remove unreacted silane molecules and dried. The product of this step will be further referred to as CA-APTS. The third step was to react the free amino groups from CA-APTS with the chlorine atoms of cyanuric chloride by immersing the membranes into an absolute ethanol solution (20 mL per membrane) containing CN in a concentration of 1 mg/mL (for 2 h at 50 °C (CA-CN)). After this step, the functionalized membranes were washed with ethanol and water to remove unreacted cyanuric chloride and soluble by-products and dried. After purification, the crown ether molecules were grafted onto CA-CN membranes through chemical bonding between the free chlorine atoms from the CN linked to the CA precursor and the free amino groups of CE (CA-CE). This reaction was performed by impregnation of the CA-CN membranes in an aqueous solution (20 mL per membrane) containing the crown ether at a concentration of 0.2 mg/mL for 2 h at 60 °C. After the completion of the reaction, the resulting CA-CE membranes were rinsed with distilled water to remove any unreacted compounds and dried. All the samples were dried prior to characterization.

### 2.3. Characterization

Fourier Transform Infrared Spectroscopy (FTIR) spectra were recorded on a Bruker Vertex 70 equipment (Bruker Optics GmbH & Co. KG, Ettlingen, Germany) in the 400–4000 cm^−1^ range with 4 cm^−1^ resolution and 32 scans. The samples were analyzed on the attenuated total reflection (ATR) module.

X-ray photoelectron spectrometry (XPS) analysis was performed on a K-Alpha spectrometer (Thermo Fisher Scientific, East Grinstead, UK) with a monochromatic Al Kα source (1486.6 eV) in a vacuum base pressure of 2 × 10^−9^ mbar. Charging effects were compensated using a flood gun, and binding energy was calibrated by placing the C1s peak at 284.8 eV as internal standard. Deconvolution of C1s signals was performed through a smart background algorithm with a convolved Gaussian–Lorentzian ratio. The pass energy for the survey spectrum was set at 200 eV, while for the high-resolution C1s spectra was 20 eV.

Thermogravimetric analysis (TGA) was performed using Netzsch TG 209 F1 Libra, NETZSCH-Gerätebau GmbH, Selb, Germany equipment, from RT to 700 °C under a nitrogen atmosphere with a heating rate of 10 °C/min.

Differential scanning calorimetry (DSC) curves were recorded on a Netzsch DSC 204 F1 Phoenix equipment, NETZSCH-Gerätebau GmbH, using a heating–cooling program from RT to 300 °C at a heating rate of 10 °C/min under a nitrogen atmosphere (20 mL/min flow rate). Based on DSC results, relative crystallinity *X_C_* was computed with the aid of the following formula:

(1) $$X_{c}=\frac{{∆H}_{melt}-{∆H}_{cryst}}{{∆H}_{100\%}}\times 100$$

where Δ*H_melt_* represents the enthalpy associated with the melting transition and Δ*H_cryst_* represents the enthalpy associated with the crystallization transition. Δ*H*_100%_ corresponds to the proposed fusion enthalpy of a perfect crystal of cellulose triacetate. For the computations implied in the determination of relative crystallinity, a value of 58.8 J/g was used according to the literature data [45].

Contact angle (CA) measurements were performed with the aid of a Drop Shape Analyzer-DSA100 from Krüss Scientific GmbH, Hamburg, Germany using water and ethylene glycol as probing liquids at room temperature by using the sessile drop method and the contact angle values were determined using the Young–Laplace equation in Advance software. Contact angle values were calculated from 20 measurements performed at each of three different positions on the membrane surface. The results are expressed as mean ± standard deviation. No inferential statistical tests were applied, as the analysis was limited to evaluating reproducibility and variability between replicate measurements.

The membranes’ performance in terms of water flux and metallic ions retention was measured at room temperature using a dead-end filtration setup. Water flux measurements were performed by passing 250 mL through a 47 mm diameter membrane disks at a transmembrane pressure of 0.8 bar. The feed solution was recirculated 5 times during the measurement for calculating and presenting the error bars. For metallic ions, retention solutions of 1 g/L for both ions were prepared. UV-Vis analysis was performed on feed solutions before and after circulation through the membrane at wavelengths of λ = 742 nm (for Cu^2+^) and λ = 392 nm (for Ni^2+^) using a Shimadzu UV-3600 UV-VIS spectrometer (Shimadzu Corporation, Kyoto, Japan) equipped with a quartz cell having a light path of 10 mm. All experiments were performed in triplicate, and the results are presented as mean ± standard deviation (SD).

The water flux (*Wf*) expressed in L·m^−2^·h^−1^ is calculated by the following equation:

(2) $$Wf=\;\frac{V}{A\cdot t}$$

where *Wf* is the water flux, *V* is the volume (L) of the feed solution, *A* is the area (m^2^) of the membrane and *t* is the time (h).

Retention efficiency (*R*%) was calculated based on the following equation:

(3) $$R\left(\%\right)=\;\frac{Cf-Cp}{Cf}\;\times 100$$

where *Cf* is the solute concentration in the feed (mol·L^−1^) and *Cp* is the solute concentration in the permeate (mol·L^−1^).

Membrane performances and water flux measurements were performed in triplicate, and the results are expressed as mean values ± standard deviation. No inferential statistical tests were applied, as the analysis was limited to evaluating experimental reproducibility and variability between replicate measurements.

Scanning electron microscopy (SEM) was performed using a Quanta Inspect F microscope (Thermo Fisher Scientific, Hillsboro, OR, USA), equipped with an energy-dispersive X-ray spectrophotometer (EDX), with the accelerating voltage being set at 30 kV.

## 3. Results

### 3.1. Structural Characterization

**Figure 2:**
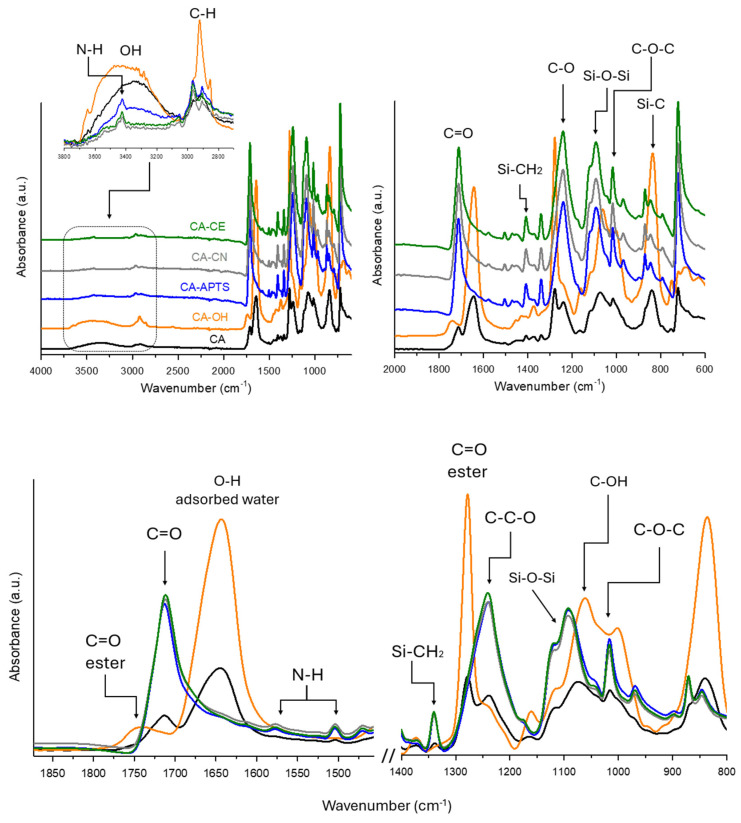
FTIR spectra of CA and its functionalized derivatives.

FTIR analysis was employed to evaluate the chemical structure of CA and its derivatives after each functionalization step, and the corresponding spectra are presented in Figure 2. The spectra of the pristine CA membrane display the characteristic absorption bands associated with its acetyl and polysaccharide backbone. The peak from 1713 cm^−1^ corresponds to the C=O stretching of acetyl groups, while the bands from 1374 cm^−1^ and 1240 cm^−1^ arise from the C–H bending and C–O stretching vibrations of the acetyl functionalities [46].

All samples display several common peaks that are characteristic for the cellulose acetate backbone such as the signal corresponding to the C–H stretching of CH_2_ and CH_3_ groups between 2800 and 3000 cm^−1^, the broad signal between 1016 and 1073 cm^−1^ that is associated with the C–O–C stretching of the pyranose ring, and the peak from 839 cm^−1^ which corresponds to the C–H and O–H bending vibrations that are characteristic of the β-glycosidic linkages between the sugar units of the cellulose backbone [47].

After the first step of the functionalization reactions, the appearance of new absorption bands and the variation in existing peaks confirm the success of the partial deacetylation reaction. Thus, the CA-OH membrane exhibits a broad O–H stretching band in the 3300–3500 cm^−1^ region, consistent with the formation of numerous hydroxyl groups [48]. Additionally, a new signal appears at 1061 cm^−1^ which corresponds to C–O stretching of hydroxyl groups and glycosidic linkages in cellulose [49]. The characteristic signal for acetyl groups from 1713 cm^−1^ shifts to 1745 cm^−1^ which corresponds to stretching of C=O from ester [45] and the peak from 1240 considerably decreases. These findings sustain the success of the deacetylation reaction.

Moreover, the peaks associated with the CA backbone become sharper and more intense after the NaOH-induced deacetylation. This effect may arise from the increased number of hydroxyl functionalities, which favors the formation of a strong hydrogen-bonds network within the polymer, as well as from residual acetate species that may remain entrapped within the membrane pores.

The spectrum of CA-APTS shows several distinctive features associated with silane grafting onto the CA-OH surface, including N-H-related absorptions bands at 3400 and in the 1504–1576 cm^−1^ region, Si-CH_2_ vibrations around 1337–1409 cm^−1^, and pronounced Si-O-Si and Si-O-C bands between 1093 and 960 cm^−1^. All these new peaks confirm the successful introduction of aminopropyl silane groups onto CA membranes [50,51].

For the CA-CN and CA-CE membranes, only small differences can be observed compared to the neat CA membrane. This is expected since the characteristic vibrations of cyanuric chloride and crown ether overlap with the strong C–O and C–O–C bands of the CA backbone in the 1200–900 cm^−1^ region. Therefore, their signals appear mainly as slight intensity changes or band broadening rather than distinct new peaks. These small modifications, although not very pronounced, are consistent with the successful introduction of the CN and CE functional groups [52].

**Figure 3:**
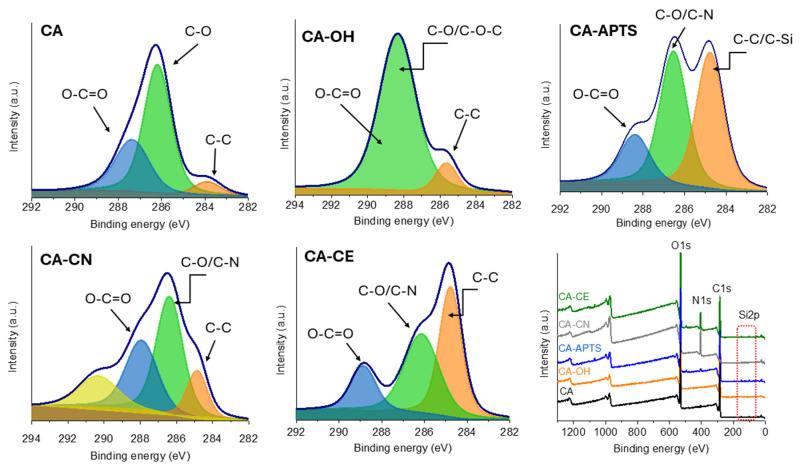
High-resolution C1s spectra of CA membranes and their functionalized derivatives.

XPS was employed to investigate the surface elemental composition of CA and its chemically modified derivatives, and the corresponding results are presented in Figure 3 and Table 1. Due to the surface-sensitive nature of the XPS technique, the elemental composition was assessed qualitatively.

Following the alkaline deacetylation stage (CA-OH), the data comprised in Table 1 show a decrease in the C–C/C–O ratio as a quantitative indicator for the removal of acetyl functional groups and the formation of a hydroxyl-rich surface in a strong basic medium. The 2.5% nitrogen recorded for this sample is attributed to adventitious surface contamination or absorbed nitrogen-containing species. For the CA–OH membrane, the residual O–C=O contribution coming from the partial deacetylation may partially overlap with the broad C–O/C–O–C region. Subsequent silanization with APTS (CA-APTS) introduced the characteristic hetero-elements Si and N and led to an increase in the aliphatic carbon contribution, confirming the grafting of silane moieties [53,54]. Additionally, the increase in nitrogen content of 10.17% sustains the grafting of the amino silane moieties. However, the N/Si ratio was not considered a direct stoichiometric indication since the elemental composition may be influenced by non-uniform silane deposition, partial hydrolysis, as well as surface orientation effects [55]. In the CA-APTS sample, the APTS structure also contains C–Si bonds. However, the C–Si contribution in the C1s spectrum may overlap with the C–C/C–H region and therefore could not be clearly separated as an individual component. Consequently, the incorporation of APTS was mainly supported by the presence of Si2p and N1s signals, while the possible C–Si contribution was considered within the low-binding-energy carbon component.

The elemental surface composition of the modified membranes shown in Table 1 is in accordance with the reaction steps undertaken for their functionalization. Thus, the variation in N 1s atomic percent values during the functionalization process is well correlated with the availability of corresponding nitrogenous functional groups, as illustrated in Figure 1.

After coupling with cyanuric chloride (CA-CN) and final attachment of the amine-terminated crown ether (CA-CE), Si remained present, demonstrating the stability of the silane anchoring layer, while N persisted throughout the functionalization sequence, consistent with nitrogen-containing linkers [52]. The decreased content of N% recorded for these two samples (CA-CN and CA-CE) can be a consequence of the partial substitution of hydrolysis of both triazine and APTS moieties as well as surface rearrangement during the purification process. The absence of chlorine on the XPS survey spectra may be related to the low amount of chlorine-containing moieties at the membrane surface and partial hydrolysis of the reactive triazine chloride groups [56].

A deeper view into the structural changes obtained after surface functionalization of CA membranes was done through the deconvolution of high-resolution C1s spectra (Figure 3) in order to sustain the above findings. Thus, neat CA deconvolutes spectrum display in three secondary peaks: the first at 284.8 eV assigned to C–C/C–H, the second one at 286.1 eV corresponding to C–O, and the third at 287.9 eV coming from the O–C=O groups as ester components. In the spectrum of CA-OH, a pronounced reduction in the O-C=O contribution after deacetylation can be observed, with subsequent increase in the contribution of C–O species [57]. Similarly, the silanization process led to an increased area for the peak assigned to C–O/C–N induced by the C–N species from attached APTES, while cyanuric chloride coupling led to an increase in the species-related carbon contributions and introduced higher-binding-energy carbon signals, consistent with the formation of triazine moieties as also demonstrated by Fatona and colleagues [58]. The final crown ether functionalization was evidenced by a considerable increase in ether-type C–O contributions while preserving only a part of C-N signals due to the possible steric hindrance effect caused by molecular volumes of the attached crown ether, thus confirming the successful covalent immobilization of the crown ether through the triazine linker. Due to the complex surface chemistry, of both CA–CN and CA–CE membranes, the C1s deconvolutions contain partially overlapping contributions from the cellulose acetate backbone, silane and triazine linker and grafted crown ether units. Therefore, the deconvolution was completed by considering the main expected carbon species and the results were interpreted qualitatively.

### 3.2. Thermal Properties

**Figure 4:**
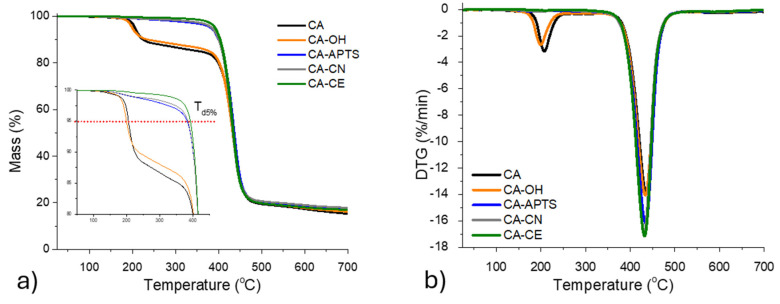
TGA (**a**) and DTG (**b**) curves of CA-based membranes.

**Figure 5:**
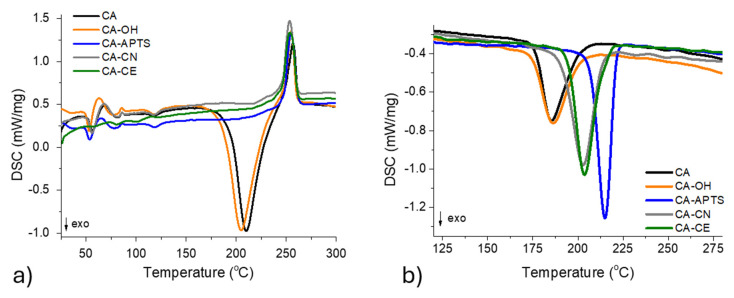
DSC thermograms of CA membrane and its functionalized derivatives. Heating (**a**) and cooling (**b**).

When it comes to the industrial water-purification process, the membranes need to withstand higher temperatures, thus the evaluation of thermal behavior is important. Additionally, it can provide valuable insights into surface chemistry and chemical modification of the samples. Thermal behavior was evaluated using TGA (and the corresponding DTG curves) as well as DSC. The corresponding thermograms are presented in Figure 4 and Figure 5 and the corresponding data in Table 2.

Thermal stability graphs revealed that all samples exhibit a major degradation step around 430 °C associated with the scission of the cellulose backbone [59]. Neat CA and the CA-OH membrane display an additional thermal event at ~200 °C which can be attributed to the removal of physically absorbed water as well as thermal-induced deacetylation [60]. Temperatures around 200 °C induce the cleavage of acetyl groups from the polymer backbone resulting in a mass loss of ~10%. Along with the functionalization reaction, the APTS, CN and CE-modified membranes display considerably higher onset degradation temperatures, with T_d3%_ values ranging from 348 to 381 °C and an increased char yield. These indicate that the incorporation of APTS, cyanuric chloride, and crown ether enhances the thermal stability of the CA matrix, likely due to the introduction of more thermally robust moieties or additional crosslinking effects [61,62].

Nevertheless, all samples exhibit close values for T_max_ between 431 and 436 °C and residual masses, implying that the primary decomposition mechanism of the cellulose backbone remains constant after functionalization.

To support the observations derived from the TGA measurements, DSC [63] was carried out as a complementary thermal analysis technique as it can provide additional insight into the thermal transitions and stability of the pristine and functionalized CA membranes (Figure 5). The CA and CA-OH samples displayed a prominent exothermal event around 210–220 °C. These findings correlate well with the TGA results highlighting the occurrence of a chemical process [64]. This conduct is consistent with the thermal-induced deacetylation or oxidation processes that take place with the release of volatile products such as acetic acid. However, previous studies concluded that, around 220 °C [65], crystallization process of the amorphous regions of cellulose acetate occur and thus these processes may overlap on the thermogram.

As functionalization reactions proceed, exothermal events are no longer present in the DSC curves of CA-APTE, CA-CN and CA-CE samples. These results correlate with the TGA, where the 10% mass loss does not occur. This suggests that additional moieties such as silane, triazine and crown ether increase thermal stability by creating a more stable layer.

The second endothermal event around 250 °C may describe a melting-like thermochemical transition of cellulose acetate domains. The maximum temperature of this peak gradually decreases after each functionalization step. The introduction of additional functional groups leads to a decreased fraction of the polymeric domain, and they are then able to undergo crystallization and formation of more rigid segments with limited mobility. This may be a consequence of multiple interactions and crosslinking processes that may take place after each grafting step. Thus, APTS leads to the formation of a silane layer that will reduce the mobility at the surface; cyanuric chloride induces rigidity while the bulky structure of crown ether will increase stability and create more sterically hindered interfacial regions.

DSC results were employed to compute the relative crystallinity (X_C_) which, in the case of cellulose acetate, can be correlated with the degree of molecular order and the influence of secondary interactions. Along with functionalization, it can be observed from Table 3 that the X_C_ value decreases. This indicates that more flexible substituents are introduced that may act as plasticizers [66,67]. The same conduct is indicated by the values for Tg. The introduction of rigid and bulky substituents leads to an increase in both X_C_ and Tg in comparison with the CA sample. In this case, the rigid triazine rings and crown ether units may increase the molecular order through strong interactions such as π-π, or the host guest reducing mobility of the macromolecular segments [68,69].

### 3.3. Surface Properties and Membrane Performances

**Figure 6:**
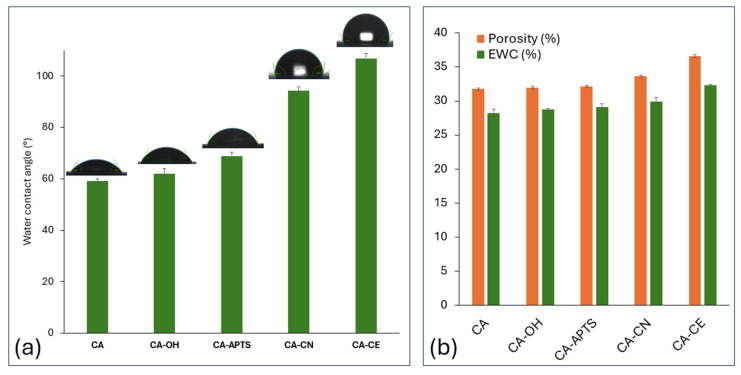
Water contact angle (**a**) and porosity and (EWC) (**b**) of functionalized CA membranes.

Water contact angle measurements were performed to evaluate the changes in surface wettability of CA and its chemically modified derivatives, and the corresponding results are presented in Figure 6a. As expected, the CA sample exhibited a hydrophilic conduct (60° WCA) as a consequence of its polar acetate and residual hydroxyl groups [70]. After treatment with sodium hydroxide (CA-NaOH), a slight increase in the contact angle can be noticed. This effect arises from the partial deacetylation process that, beside acetate group removal, sustained a redistribution of surface hydroxyl groups, which may consequently reduce wettability. Additionally, surface rearrangements may take place, exposing fewer polar domains or morphological heterogeneity of the membrane surface. Subsequent functionalization with APTS resulted in a further increase in the contact angle (~70°) which can be attributed to the incorporation of hydrophobic propyl and silane moieties [70].

After the modification of CA-APTES with cyanuric chloride (CA-CN), the contact angle significantly increased to approximately 95°, indicating a transition toward a more hydrophobic surface [58]. This is a consequence of the introduction of aromatic triazine structures and their reduced surface polarity. Finally, crown ether-functionalized CA (CA-CE) shows the highest contact angle vales (~105°), denoting an enhanced hydrophobic character [71]. This may be due to steric hindrance, increased surface roughness, and due to the nonpolar character of crown ether units. At the same time, the rigid and bulky triazine and crown ether moieties can create a surface layer that protects the underlying polar groups present on the cellulose backbone.

In contrast, the porosity and EWC values (Figure 6b) remain relatively high for all membranes and even show a slight increase for CA-CN and CA-CE in comparison with the CA membrane [72]. This suggests that, although the outer surface becomes more hydrophobic, the functionalization steps do not alter the density and the internal structure of the membranes. Additionally, the pore volume and the capacity of the membranes to absorb water are preserved. Overall, these results show that the surface and the bulk of the membranes have a distinct conduct. Although the CN and CE-modified membranes have a hydrophobic surface, they maintain high porosity and a good ability to absorb water.

### 3.4. Membrane Performances

The metallic ion rejection performance was evaluated over five consecutive filtration cycles against Ni^2+^ and Cu^2+^ ions. These metallic species were considered, as it was previously demonstrated that divalent cations can interact with the polymeric matrix through various interactions such as electrostatic and hydrogen bonds, increasing the removal efficiency [73,74]. Since pH and ionic strength can strongly affect metal ion speciation and competitive ion transport, their influence should be systematically investigated in future works. In the present study, the experiments were conducted under fixed conditions to primarily assess the effect of crown ether functionalization on membrane performance.

**Figure 7:**
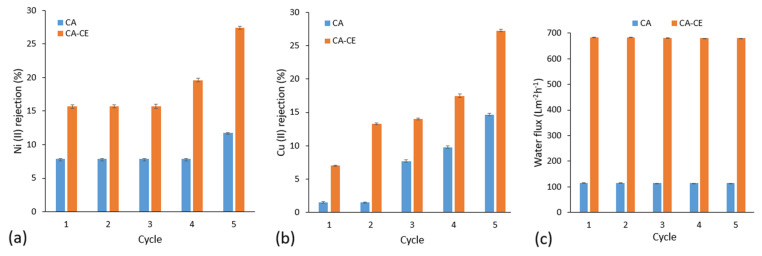
Performance of CA and CA-CE membranes in terms of Ni(II) rejection (**a**), Cu(II) rejection (**b**), and water flux (**c**) over five filtration cycles.

As shown in Figure 7, the incorporation of crown ether moieties leads to a substantial improvement in retention performance as the results highlight a clear distinction between the CA and the CA-CE membranes. For Ni^2+^, the CA-CE membranes exhibit a progressive increase from approximately 15% in the first cycle to ~30% in the fifth cycle, whereas the CA membranes maintain consistently low rejection values below 10%. A similar trend is observed for Cu^2+^, where the CA-CE membranes achieve rejection efficiencies almost three times higher than those of CA, reaching ~27% in the final cycle. During the fifth cycle, the CA-CE membranes showed an increase in rejection that may take place due to complexation interactions occurring between the crown ether groups grafted onto the CA membranes’ surface and Ni^2+^ and Cu^2+^ ions [75]. This is due to progressive membrane conditioning during filtration. Repeated exposure to the aqueous solution may improve wetting of the functionalized membrane and stabilize water transport pathways, thereby increasing pore accessibility and flux.

The retention efficiency of the crown ether units is strongly influenced by the ion radius which will exert strong binding interactions with a cavity with a suitable dimension [76]. Since Ni^2+^ and Cu^2+^ have smaller ionic radii than the typical cavity preference of the 15-crown-5 ether, the retention of the metallic species takes place through combined chemical and membrane-related effects. The grafter macrocyclic ligands provide oxygen-rich coordination sites that can interact with the metallic ions at the membrane surface while neighboring functional groups of the functional membranes such as silane and triazine may also contribute to multi-site interactions atoms [76,77].

The improved Ni^2+^ and Cu^2+^ retention of CA-CE is attributed to combined effects of crow-ether-assisted surface interactions, neighboring functional groups and modified membrane transport properties. However, adsorption isotherms and quantitative crown ether loading were not determined in this study and will be addressed in future work.

Complexation involving one or multiple CE units may lead to localized crosslinking or densification of the functionalized surface layer. Such structural rearrangements can enhance the barrier properties of the membrane during repeated operation, therefore contributing to the pronounced increase in rejection in the later cycles [44].

Since studies addressing cellulose acetate membranes covalently functionalized with crown ether for Ni^2+^ and Cu^2+^ retention are limited, the present system was compared with representative systems (Table 4) containing polymers and crown ether units developed for heavy metal ion removal.

Additionally, water flux for CA and CA-CE membranes was evaluated and the corresponding results are presented in Figure 7. The CA membrane displays an initial flow of ~120 Lm^−2^h^−1^ with a small decrease over the five cycles. This is a consequence of the gradual compression of the polymer chains within the porous layer under hydraulic pressure, which stabilizes the internal structure of the membrane over repeated operation [44]. The CA-CE membranes display a remarkably higher water flux, maintaining values around 620–650 Lm^−2^h^−1^ throughout all cycles. The stability of the water flux indicates that CE grafting not only improves water transport but also contributes to structural integrity and hinders the fouling during repeated operation.

The higher water flux observed for the functionalized membranes indicates that permeability was influenced not only by surface wettability. The progressive modification and purification steps may have also improved pore accessibility, promoted membrane conditioning and included structural rearrangements that facilitated water transport.

Although the CA-CE membrane exhibits a higher apparent contact angle than the neat CA membrane, an increased flow rate was observed. This indicates that water transport is not governed only by the surface wettability. The contact angle mainly reflects the interaction between water and the exterior membrane surface, whereas the flow rate is strongly influenced by the internal structure of the membrane, including pore accessibility, water transport pathways, swelling behavior and morphological changes induced by functionalization. Therefore, the higher flow rate of CA-CE may be attributed to improved pore accessibility and membrane conditioning after modification, rather than hydrophobicity itself.

Overall, the enhancement in both permeability and metal ion rejection demonstrates the synergistic effect of CE functionalization, confirming that the developed CA-CE membranes exhibit good performance and long-term stability compared to unmodified CA membranes.

The retention studies were performed using single-solute Cu^2+^ and Ni^2+^ solutions to evaluate the effect of crown ether functionalization under controlled conditions. Building on these results, future work will consider multicomponent systems and simulated wastewater matrices in order to further assess ion selectivity and performance under conditions closer to practical water treatment applications.

XPS was further used to confirm the presence and surface retention of Cu^2+^ and Ni^2+^ ions on CA and CA-CE membranes after the adsorption experiments. As shown in Table 5, pristine CA membranes exhibit only a trace amount of copper (0.09 at.%), indicating weak interaction between the polymer matrix and metal ions. In contrast, CA-CE membranes show higher metal atomic percentages, with Cu reaching 0.12 at.% and Ni 1.47 at.%, demonstrating enhanced metal ion retention upon crown ether functionalization.

### 3.5. Surface Morphology

**Figure 8:**
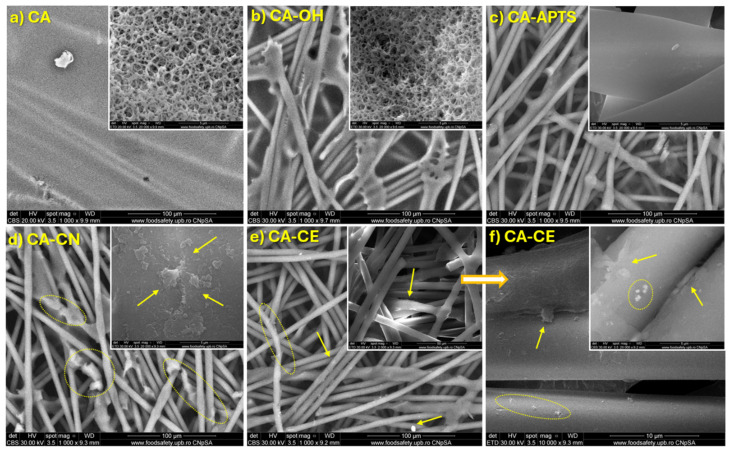
SEM micrographs of CA and its functionalized derivatives.

Surface morphology and roughness of CA and functionalized derivative membranes were evaluated through SEM, and the corresponding micrographs are presented in Figure 8. The pristine CA membrane exhibits a smooth surface with uniform and well-defined pores. After deacetylation with NaOH, the CA-OH sample displays a slightly rougher surface with more defined fibers. Additionally, NaOH treatment opens the dense surface layer and removes internal additives from the commercial CA membrane, creating more open spaces and increasing the overall porosity and roughness. It can clearly be observed that along with the functionalization process, fiber morphology changes accordingly after each step.

APTS functionalization produces a distinct morphology characterized by a smoother and more continuous coating on the fiber surfaces. In the case of the CA-CN sample, the bulky domains are consistent with surface grafting of triazine rings and partial crosslinking, as previously reported for cyanuric chloride-modified membranes [62,83]. The resulting increase in surface roughness and functionality indicates a high density of reactive sites. CA-CE fibers display increased roughness, localized surface protrusions and heterogeneous domains, attributed to the covalent grafting and partial aggregation of bulky crown ether moieties [75] (Figure 8e,f). These surface features are attributed to the attachment of bulky macrocyclic crown ether moieties, which commonly form non-uniform surface coatings due to their size, hydrophobic character, and tendency to aggregate [84]. Overall, a progression from smooth (CA) to increasingly rough, chemically active surfaces (CA-CN, CA-CE) is observed, confirming the success of the functionalization reaction. Moreover, the morphological changes correlate with the improvements in metal ion affinity and retention.

## 4. Discussion

The retention of heavy metal ions represents a continuous challenge in the current context of concerns for a clean environment. If for large quantities of heavy metals we have the possibility to use ion exchangers, regardless of the nature of the ions, for small quantities, the solutions are quite limited [85,86]. The use of crown ethers for ion complexation is an elegant solution to this problem [87,88,89]. Their use is limited by their immobilization on particles or membranes to carry out the separation process. In the context of the circular economy, the use of ‘green’ polymers has led to an increase in research in this field, one of the main candidates being cellulose acetate due to its versatility in the synthesis of polymeric membranes [90,91]. The retention capacity of cellulose acetate for heavy metal ions can be explained by its ability to complex ions with the help of free electrons from acetyl groups [92]. The problem with its use in the membrane form is that multiple recirculation steps are required to increase the efficiency of the process [93]. The functionalization of cellulose acetate membranes with crown ether has been successfully reported for Gd(III) retention in aqueous solutions [44]. In this case, the crown ether was immobilized by means of the APTES and glutaraldehyde linker molecules. Also, for Gd(III) retention, cellulose acetate membranes functionalized with calmagite were used, in this case the membrane having the advantage that the membrane changes its surface color as the separation occurs [59]. Crown ethers immobilized on cellulose acetate by means of ethanolamine were also used for the retention of Ca^2+^ ions [52]. Also, the binding was achieved with the help of the APTES and glutaraldehyde linker molecules. Cellulose acetate was functionalized with Rhodamine B hydrazide for Cu(II) retention to optimize its detection, with the maximum efficiency of retention at the detection of solutions with concentrations in the range of 0–100 ppm of CuCl_2_ solution [94]. By preparing CA composite membranes with montmorillonite, a separation efficiency of 60 mg/g Cu(II)/membrane weight was obtained for a concentration of 5% montmorillonite in CA [95]. Other effective fillers for metal retention using cellulose acetate membranes are hydroxyapatite [96,97,98], magnetic particles [99,100], carbon nanotubes [101] or layered double hydroxide [102].

The use of cyanuric chloride for linking crown ethers represents another advantage reported in this work, given the fact that after the first substitution of a chlorine atom, the aromatic ring is deactivated and the crosslinking effect during membrane functionalization is avoided. Also, the immobilization of crown ethers onto cellulose acetate opens the possibility of retention of heavy metal ions for a wide range of other elements.

Although the CA-CE membrane showed improved Ni^2+^ and Cu^2+^ retention compared with neat CA, the rejection efficiencies remain moderate when considered from the perspective of practical water treatment. Therefore, the present system should be considered as a proof-of-concept functional membrane platform rather than an optimized treatment material. Further studies are required to improve retention efficiency and validate membrane performance under more realistic water-treatment conditions.

## 5. Conclusions

In this work, a stepwise functionalization strategy was successfully applied to cellulose acetate membranes to achieve the covalent immobilization of crown ether moieties via a cyanuric chloride linker. FTIR and XPS results confirmed the progressive introduction of hydroxyl, silane, triazine, and crown ether functionalities, demonstrating the effectiveness and stability of the surface modification. Thermal analyses further indicate that the functionalized membranes exhibit improved thermal stability while preserving the integrity of the cellulose backbone.

Crown ether functionalization led to a significant enhancement in membrane performance such as a substantial increase in water flux and excellent operational stability. The CA-CE membranes showed higher retention of Ni^2+^ and Cu^2+^ ions, reaching ~30% and ~27%, respectively, after five filtration cycles, compared with values below 10% for pristine CA, indicating strong affinity and stable metal coordination at the membrane surface.

These results demonstrate that crown ether immobilization is an effective approach for simultaneously enhancing permeability and heavy metal ion retention in cellulose acetate membranes, highlighting their potential for advanced water purification applications.

## Figures and Tables

**Table 1:** Elemental composition and quantification of surface analysis from XPS data.

Sample	C at.%	O at.%	N at.%	Si at.%	C–C/C–O
CA	61.53	38.47	-	-	0.36
CA-OH	62.17	34.62	2.51	-	0.11
CA-APTS	46.06	43.02	10.17	0.76	1.02
CA-CN	69.45	28.46	0.62	1.47	0.32
CA-CE	68.81	28.68	0.93	1.58	1.05

**Table 2:** TGA-derived thermal properties of CA membranes.

Sample	T_d3%_ (°)	T_d5%_ (°)	T_max_ (°) from DTG		Residual Mass (%)
**CA**	199.8	205.9	206.4	435.8	15.3
**CA-OH**	193.2	199.4	199.9	434.0	16.2
**CA-APTS**	348.0	382.4	434.4		17.3
**CA-CN**	362.1	400.8	431.3		17.9
**CA-CE**	381.3	392.4	432.7		17.0

**Table 3:** DSC parameter for CA and functionalized derivatives.

Sample	T_max1_ (°C)	Δ*H*_1_ (J/g)	T_melt_ (°C)	Δ*H_melt_* (J/g)	T_cryst_ (°C)	Δ*H_cryst_* (J/g)	X_C_ (%)	Tg (°C)
**CA**	210.2	211.7	256.3	46.38	185.4	36.57	16.68	61.3
**CA-OH**	205.1	242.6	255.1	43.51	186.1	35.33	13.91	57.1
**CA-APTS**	-	-	255.2	55.41	215.0	48.62	11.55	57.7
**CA-CN**	-	-	253.4	61.22	202.8	49.41	20.09	60.0
**CA-CE**	-	-	253.5	60.23	203.5	49.13	18.88	64.3

**Table 4:** Crown ether-modified materials for heavy metal ion retention.

Membrane	Method/Test Conditions	Target Ions	Performance	Ref.
Cellulose modified with dibenzo-18-crown 6	Batch adsorption	Cd^2+^, Zn^2+^, Ni^2+^, Pb^2+^, Cu^2+^	High removal efficiency depending on surface chemistry	[78]
Dibenzo-18-crown-6 crown ether-modified starch	Batch adsorption	Cd^2+^, Zn^2+^, Ni^2+^, Cu^2+^	After four filtration cycles, the adsorbent demonstrates high recyclability and high adsorption efficiency.	[79]
4′-aminobenzo-15-crown-5 ether functionalized hydrochar	Batch adsorption	Pb^2+^, Cu^2+^	The adsorption capacities from 10.15 to 16.86 mg∙g^−1^ for Cu^2+^ and from 8 to 11.93 mg∙g^−1^ for Pb^2+^	[80]
Dibenzo-18-crown-6-grafted bamboo pulp aerogel	Batch adsorption	Pb^2+^, Cu^2+^, Cd^2+^	Maximum Pb^2+^ adsorption capacity of 129.15 mg/g; adsorption of Pb^2+^, Cu^2+^, and Cd^2+^ followed mainly pseudo-second-order kinetics	[81]
Polyacrylonitrile dibenzo-18-crown-6 electrospun nanofibers	Batch adsorption	K^+^, Ba^2+^, Na^2+^, Li^+^	Maximum K^+^ adsorption capacity of 0.37 mmol/g; selectivity sequence K^+^ > Ba^2+^ > Na^+^ ≈ Li^+^; only ~10% capacity loss after four adsorption–desorption cycles	[82]

**Table 5:** Surface elemental composition (at.%) of CA and CA-CE membranes after Cu^2+^ and Ni^2+^ retention determined by XPS.

Sample	C at.%	O at.%	N at.%	Si at.%	Cu at.%	Ni at.%
CA-Cu	53.62	45.23	1.06	-	0.09	-
CA-CE-Cu	69.13	28.08	1.5	1.17	0.12	-
CA-Ni	50.09	42.77	3.53	2.29	-	1.31
CA-CE-Ni	70.27	25.38	0.75	2.12	-	1.47

## Data Availability

The original contributions presented in this study are included in the article. Further inquiries can be directed to the corresponding author.
